# Molecular detection of canine viral infectious diseases in China: an investigation from 2018 to 2024

**DOI:** 10.3389/fvets.2025.1709294

**Published:** 2026-01-06

**Authors:** Caihong Liu, Yalei Chen, Ningning Cui, Yihang Yang, Hangtian Ding, Hongchao Wu, Yuxiu Liu, Kegong Tian, Xingang Xu

**Affiliations:** 1 College of Veterinary Medicine, Northwest A&F University, Yangling, Shaanxi, China; 2 National Research Center for Veterinary Medicine, Luoyang, Henan, China; 3 Luoyang Huizhong Biotechnology Co. Ltd., Luoyang, Henan, China

**Keywords:** Canine viral infectious disease, molecular detection, phylogenetic analysis, prevalence, serological

## Abstract

To analyze the prevalence of canine viral diseases in China, including canine parvovirus type 2 (CPV-2), canine coronavirus (CCoV), canine distemper virus (CDV), canine herpesvirus type 1 (CHV-1), canine parainfluenza virus (CPIV), canine influenza virus (CIV), canine respiratory coronavirus (CRCoV), canine adenovirus 2 (CAV-2), canine adenovirus 1 (CAV-1), and canine rotavirus (CRV), a total of 2,492 samples from dogs in 22 provinces were tested between 2018 and 2024. The results showed that 1,236 dogs (49.6%) tested positive for one or more pathogens, CPV-2 (30.6%) being the most commonly detected, with CPV-2c being the most common genotype. The prevalence of CPV-2, CCoV, CDV, and CHV-1 varied significantly depending on the season, geographical location, and age, with young dogs (<6 months) being more susceptible to infection. The positive rates of CPV-2 and CDV were significantly higher in unvaccinated dogs than in vaccinated ones, whereas infections with CCoV, CPIV, CAV-2, and CAV-1 were not strongly associated with vaccination status. In the serological survey, the protective rates of 398 vaccinated dogs to CPV-2, CDV, CAV-1, CAV-2 and CPIV were 76.9, 72.1, 84.4, 85.7 and 49.0%, respectively. The emergence of a distinct phylogenetic clade of the CPIV F gene may contribute to the reduced protective efficacy against CPIV. Overall, these findings reveal the complex epidemiology of ten canine viral pathogens in China, highlighting the critical need for targeted prevention strategies and more effective vaccine development.

## 1 Introduction

The growing population of pet dogs in China has raised concerns about the spread of canine infectious diseases (1). Canine distemper virus (CDV) and canine parvovirus type 2 (CPV-2) are the leading causes of death in dogs due to their high morbidity and mortality rates, and they also pose a serious threat to wildlife (2). Canine rotavirus (CRV), a non-enveloped, double-stranded RNA virus, typically causes moderate gastroenteritis but can lead to severe and fatal enteritis in puppies (3). Canine adenovirus (CAV), a non-enveloped, double-stranded DNA virus, includes two genotypes: CAV-1, which causes infectious canine hepatitis (ICH), and CAV-2, which is associated with infectious tracheobronchitis (ITB) and enteritis (4–6) Canine coronavirus (CCoV), a large, enveloped, single stranded RNA virus, generally causes mild diarrhea in dogs, with high morbidity and low mortality (7, 8). Canine respiratory coronavirus (CRCoV), an enveloped virus containing a non-segmented, positive-sense, single-stranded RNA genome, is considered one of the primary causative agents of canine infectious respiratory disease (CIRD) (9, 10). Canine herpesvirus type 1 (CHV-1) is a double-stranded DNA, enveloped α-herpesvirus (11). Infection with CHV-1 in puppies aged one to 2 weeks can lead to a generalized necrotizing, hemorrhagic disease, whereas asymptomatic or mild, self-limiting infections are more common in puppies older than 3 weeks and adult dogs (11). Canine parainfluenza virus (CPIV), an enveloped, negative-strand RNA virus (12), generally causes mild or no respiratory signs in dogs. Nonetheless, some cases involving serous nasal discharge, mild pharyngitis, and tonsillitis associated with CPIV have also been reported (13). More severe respiratory illness may occur when CPIV infection is complicated by coinfection with other viruses or bacteria (14). Influenza A virus is an enveloped, negative-sense, single-stranded RNA virus, which has a complex natural history and infects a wide range of host species (15, 16). Canine influenza virus (CIV) has been circulating in China for over a decade (17). With the growing number of pet dogs in China, there is increasing concern over the potential for zoonotic transmission (1, 18). CAV-2, CDV, CPIV, CHV-1, CIV, and CRCoV are generally considered the primary pathogens responsible for canine infectious respiratory disease complex (CIRDC) in dogs (19). To further supplement the data on the epidemiology and seroprevalence of canine viral infections in China. The present study investigated the prevalence of ten major canine viruses (CPV-2, CCoV, CDV, CPIV, CHV-1, CIV, CRCoV, CAV-2, CAV-1, and CRV) by nucleic acid detection in 2,492 samples collected from dogs across 22 provinces in China between 2018 and 2024. In addition, gene sequence analyses of CPV-2 and CPIV, as well as serological analyses of CDV, CPV-2, CAV-1, CAV-2, and CPIV, were performed to provide comprehensive epidemiological insights into the canine population.

## 2 Materials and methods

### 2.1 Sample collection

**Figure 1:**
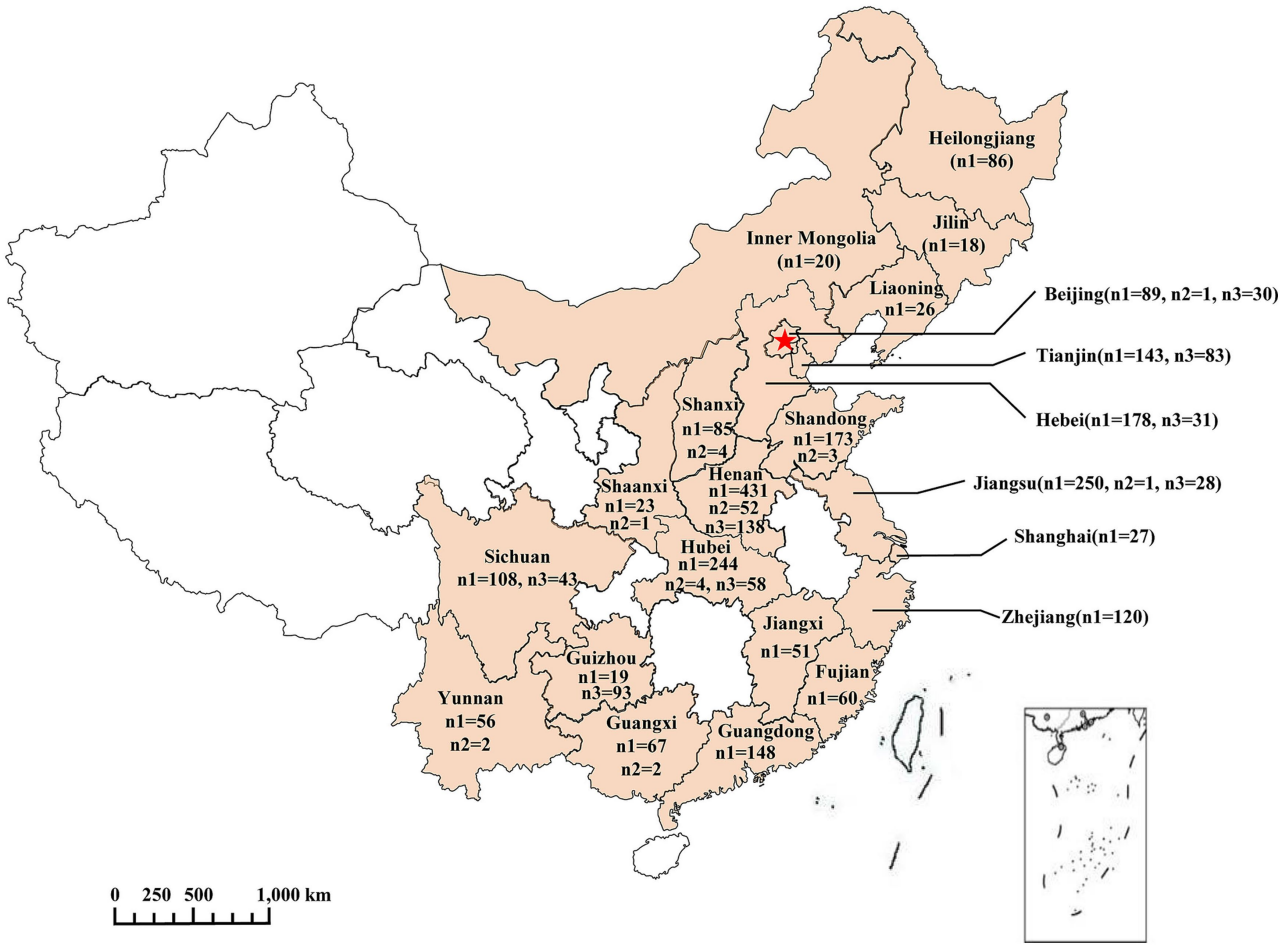
Locations of sample collection. The area of light orange in the map represents the sampled provinces. n1, number of dogs sampled for swabs; n2, number of deceased dogs from which tissue samples were collected; n3, number of serum samples. Red star, Beijing, the capital of China.

Between January 2018 and December 2024, samples were collected from veterinary clinics across 22 provinces in China (Figure 1). All samples were collected as part of the standard diagnostic procedure after obtaining informed owner consent. Clinical monitoring data were recorded for each animal, and all samples were handled in accordance with ethical guidelines. From each of 2,422 dogs exhibiting clinical signs of respiratory or gastrointestinal disease, ocular, nasal, and anal swabs were collected. In addition, tissue samples including lung, trachea, or intestines were collected from 70 dogs that died after exhibiting similar clinical manifestations. Furthermore, between 2020 and 2024, serum samples were collected from 504 dogs in 8 provinces (Figure 1).

### 2.2 PCR amplification of samples

Primers for CPV-2, CCoV, CDV, CPIV, CHV-1, CIV, CRCoV, CAV, and CRV were listed in Table 1. Nucleic acid was extracted using the Viral Nucleic Acid Extraction Kit II (Geneaid, China), following the manufacturer’s instructions. The extracted nucleic acid served as the template for one-step RT-PCR (TransGen Biotech, China) to detect CCoV, CDV, CPIV, CIV, CRCoV, and CRV. Meanwhile, CPV-2, CHV-1, and CAV were detected using PCR with Premix Taq (Ex Taq Version 2.0 plus dye; Takara, Japan). The RT-PCR reaction volume was in 15 μL, which included 7.5 μL of 2 × One-Step Reaction Mix, 0.3 μL of One-Step Enzyme Mix, 0.75 μL of each primer (10 μM), 1.5 μL of template, and 4.2 μL of ultrapure water. The RT-PCR reaction are as follows: 45 °C for 30 min, 98 °C for 3 min, followed by 30 cycles of 95 °C for 30 s, 56 °C for 30 s, 72 °C for 60 s, and 72 °C for 10 min. The PCR reaction volume was in 15 μL, which included 7.5 μL of Premix Taq, 0.75 μL of each primer (10 μM), 1.5 μL of template, and 4.5 μL of RNase-free water. The PCR reaction included an initial denaturation step at 98 °C for 3 min followed by 30 cycles of 95 °C for 30 s, 56 °C for 30 s, 72 °C for 60 s, and 72 °C for 10 min. Negative controls (H₂O) and positive controls (virus stocks from cell culture) were included in each test. Positive controls consisted of either virus stocks from cell culture [for CDV, CPIV, and CPV-2 (20–22)] or plasmid constructs containing the target gene (for CAV-1, CAV-2, CCoV, CHV-1, CIV, CRCoV, and CRV).

**Table 1:** The PCR amplification primers of major canine viruses.

Primer name	Primer sequences	Product/bp
CPV-2-F	5′-GCACATCAAGATACAGGAAG-3′	800 bp (20)
CPV-2-R	5′-CCTTAACATATTCTAAGGGCAA-3′	
CCoV-F	5′-CACACATTCTGATGGAGACG-3′	372 bp (20)
CCoV-R	5′-AGACCTCCTAGCCAAGAACC-3′	
CDV-F	5′-ACTTGCAGGTGTAGCTTTAGG-3′	324 bp (20)
CDV-R	5′-AATAGGATCACGTAAACTCGG-3′	
CHV-1-F	5′-GAGAACCCTTTGGAATGAAG-3′	713 bp
CHV-1-R	5′-TAAGAAACGAGGACACTCCA-3′	
CPIV-F	5′-GAAACGATTAGGAACCAGTTGAT-3′	475 bp (20)
CPIV-R	5′-GCACTTATCTGGGTATTGAAAG-3′	
CIV-F	5′-TTCTAACCGAGGTCGAAACGTA-3′	229 bp
CIV-R	5′-AAGCGTCTACGCTGCAGTCCTCGC-3′	
CRCoV-F	5′-GCAATGCTGGTTCAGAAG-3′	442 bp
CRCoV-R	5′-GTTGGTATAGGTGAGCA-3′	
CAV-F	5′-CGCGCTGAACATTACTACCTTGTC-3′	CAV-1 520 bp (20);CAV-2 1,030 bp
CAV-R	5′-CCTAGAGCACTTCGTGTCCGCTT-3′	
CRV-F	5′-TATGATGCAGCGTTGC-3′	457 bp
CRV-R	5′-ATACTTGCCACCATTTC-3′	

### 2.3 Virus neutralization and hemagglutination inhibition assays

Neutralization assay were performed to detect the neutralization antibody of CDV, CAV-1, CAV-2 and CPIV. Blood samples collected from dogs were centrifuged at 5000 rpm for 5 min and heat-inactivated at 56 °C for 30 min. Serial two-fold dilutions of the serum, starting from 1:2, were prepared and added to 96-well microtiter plates. Each dilution was pre-incubated with an equal volume of the corresponding virus strain (containing 10^2^TCID₅₀ units) for 1 h at 37 °C. Subsequently, 2.5 × 10^4^ Vero or MDCK cells were added to each well. The plates were then incubated at 37 °C in a humidified atmosphere containing 5% CO₂ for 4–5 days. Neutralizing antibody titers were determined using the Reed-Muench method.

Hemagglutination inhibition (HI) assays were conducted to evaluate serum antibody titers against CPV-2. Serial two-fold dilutions of serum, ranging from 1:2 to 1:4096, were prepared in 96-well V-bottom microtiter plates. Next, 8 HA units of CPV-2 were added to each well, and the plates were incubated at 37 °C for 30 min. Following this, 0.025 mL of a 1% suspension of porcine red blood cells was added to each well. The plates were then incubated at 4 °C for 90 min. The HI titer was defined as the highest serum dilution that completely inhibited hemagglutination.

### 2.4 Phylogenetic analysis of CPIV and CPV-2

To investigate CPIV and CPV-2 genotypes circulating in China, samples were selected for sequencing based on clear PCR band quality and geographical and temporal representation. Ocular, nasal, and anal swabs from two dogs with severe respiratory signs (paroxysmal cough and retching), diagnosed with CPIV by RT-PCR, were used for sequencing of the fusion (F) gene. The primers used were as follows: CPIV-4-F: 5′-GACATTCGTAAATACCTATGGATTC-3′, CPIV-4-R: 5′-GCTTGAAATTGATGTTATATTACATCCA-3′, CPIV-5-F: 5′-ATTCCAACAAATGTCCGGCAACT-3’, CPIV-5-R: 5′-GCCAATTGAGTGATGGTGAATCT-3′, CPIV-6-F: 5′-TGACATGTACAAATGTGTGAGTCTGCAGC-3′, CPIV-6-R: 5′-CCACGAGCAGTTCTGTTCTAGCT-3′. The sequencing procedure was performed as previously described (21). 30 samples collected from various provinces and at different time points were sequenced. The primers and sequencing method followed that have been detailed described elsewhere (23). Phylogenetic analysis of CPV-2 and CPIV were conducted by maximum likelihood method in MEGA 5.1 (bootstrap replicates = 1000). The phylogenetic tree of VP2 gene was annotated using the Interactive Tree of Life (iTOL) software,^1^ an online tool for the display and annotation of phylogenetic trees.

### 2.5 Data analysis

Statistical comparisons of prevalence by season, geographical location, age group, and immunization status were performed using the chi-square (*χ*^2^) test. A *p*-value <0.05 was considered statistically significant.

## 3 Results

### 3.1 Prevalence of ten viral infections in dogs

**Figure 2:**
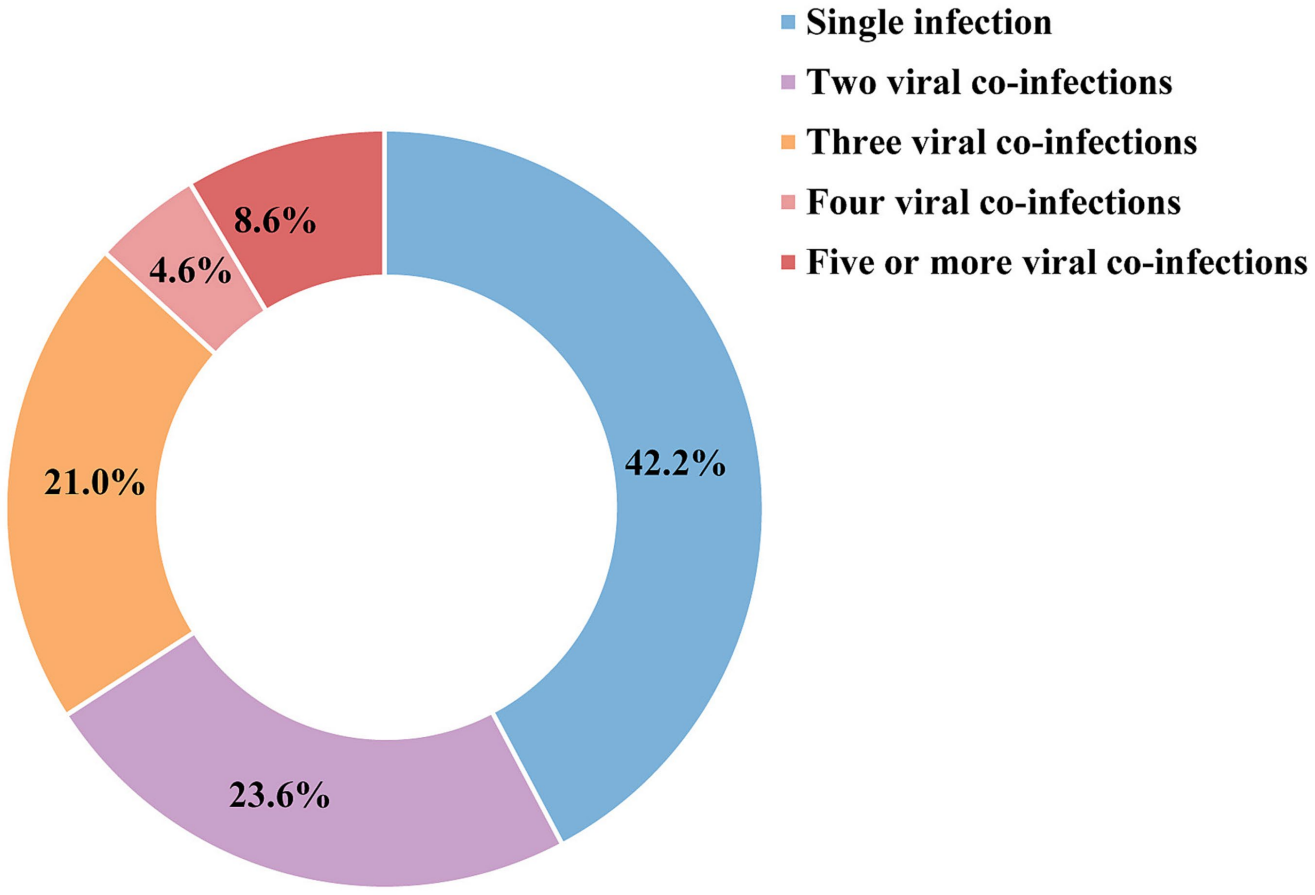
Infection patterns of dog viral diseases.

**Figure 3:**
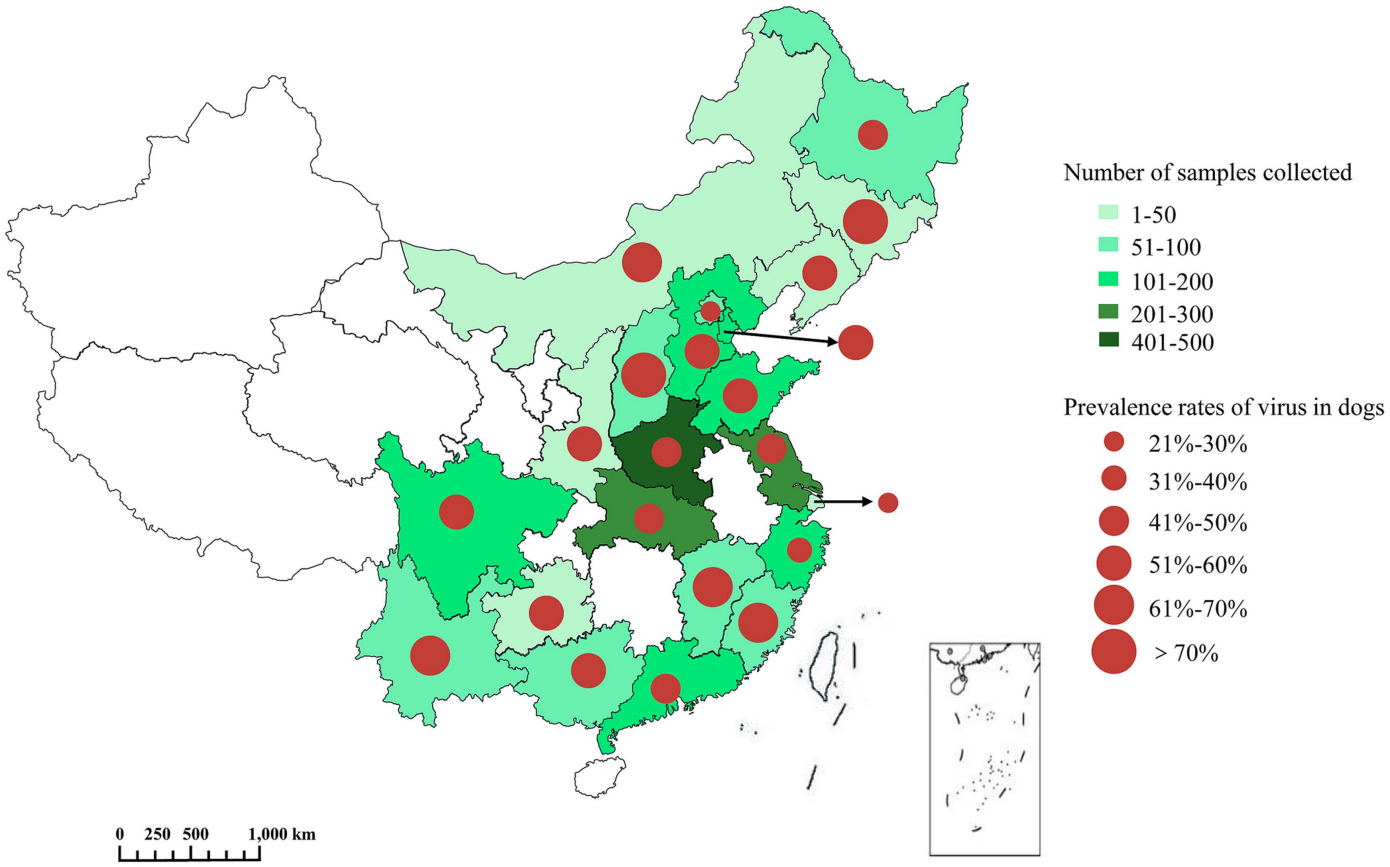
Prevalence rates of virus in dogs across China in 2018–2024. Provinces with sampling points marked green, and the sampling quantity is reflected by the color depth. The symbol size of red dots was used to describe the size of prevalence.

**Figure 4:**
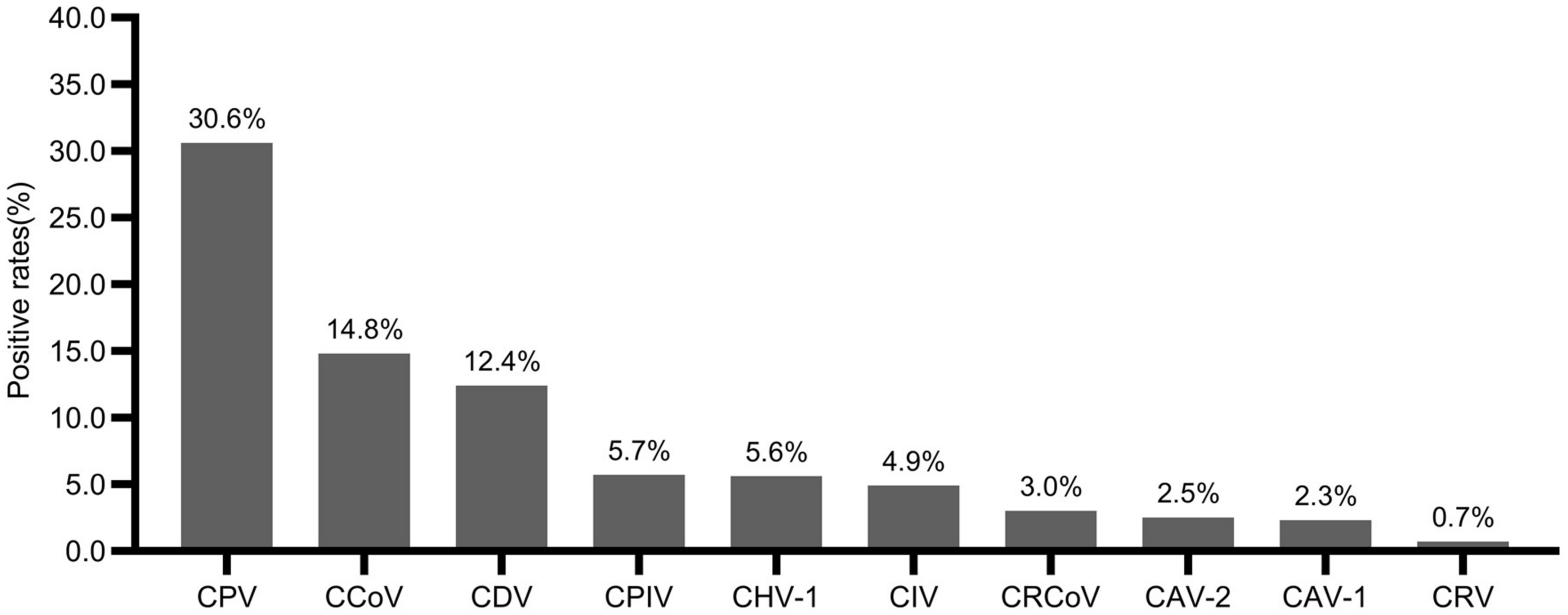
Positive rates of CPV-2, CCoV, CDV, CPIV, CHV-1, CIV, CRCoV, CAV-2, CAV-1 and CRV of dogs in China during 2018–2024.

A total of 2,422 swabs and 70 tissues samples were collected from 2,492 animals in 22 provinces and screened for ten canine viral pathogens. These provinces are geographically distributed from Heilongjiang in the north to Guangdong in the south. Herein, 10 pathogens, including CPV-2, CCoV, CDV, CPIV, CHV-1, CIV, CRCoV, CAV-2, CAV-1, and CRV, were detected from 2018 to 2024. 1,236 (49.6%) dogs were tested positive for one or more pathogens, and 42.2% (522/1,236) were infected with a single virus, while 57.8% (714/1,236) had mixed infections. As shown in Figure 2, the co-infection rates are as follows: 23.6% for two viruses, 21.0% for three viruses, 4.6% for four viruses, and 8.6% for five or more viruses. The prevalence of dogs infected with one or more viruses varied significantly among provinces, ranging from 27.8 to 83.3% (Figure 3). Notably, the prevalence rate were comparatively lower in major cities such as Beijing and Shanghai. In terms of pathogen-specific prevalence, CPV-2 (30.6%, 763/2,492), CCoV (14.8%, 369/2,492), and CDV (12.4%, 309/2,492) were the most frequently detected, followed by CPIV (5.7%, 143/2,492), CHV-1 (5.6%, 139/2,492), CIV (4.9%, 122/2,492), CRCoV (3.0%, 75/2,492), CAV-2 (2.5%, 62/2,492), CAV-1 (2.3%, 57/2,492), and CRV (0.7%, 17/2,492) (Figure 4).

### 3.2 Seasonality of ten viral infections

The present study used statistical comparisons of prevalence by season, geographical location, age, and immunization status. The exposure rates of CPV-2, CCoV, CDV, CPIV, CHV-1, CIV, CRCoV, CAV-2, and CRV were significantly associated with season (Tables 2, 3). For CAV-1, there was no significant difference in the exposure rates of different seasons. The prevalence of CCoV (21.6%), CPIV (13.5%), CIV (14.0%), CAV-2 (6.1%), and CRV (1.8%) in winter (December to February) was higher than in spring (March to May), summer (June to August), and autumn (September to November). In contrast, the prevalence of CDV (16.4%) and CHV-1 (7.7%) was higher in spring than in winter, summer, and autumn. CRCoV (4.4%) was more frequently detected in autumn. For CPV-2, the prevalence in spring (38.8%) and winter (38.6%) was higher than in summer (25.8%) and autumn (20.6%).

**Table 2:** Prevalence of main canine viruses infection in different categories.

Factors	Category	No.	No. of positive dogs (positive %)									
			CPV-2	CCoV	CDV	CPIV	CHV-1	CIV	CRCoV	CAV-2	CAV-1	CRV
Season	Spring	780	303 (38.8%)	129 (16.5%)	128 (16.4%)	37 (4.7%)	60 (7.7%)	24 (3.1%)	30 (3.8%)	8 (1.0%)	13 (1.7%)	5 (0.6%)
	Summer	702	181 (25.8%)	45 (6.4%)	61 (8.7%)	22 (3.1%)	14 (2.0%)	3 (0.4%)	6 (0.9%)	15 (2.1%)	15 (2.1%)	2 (0.3%)
	Autumn	616	127 (20.6%)	110 (17.9%)	65 (10.6%)	31 (5.0%)	36 (5.8%)	40 (6.5%)	27 (4.4%)	15 (2.4%)	21 (3.4%)	3 (0.5%)
	Winter	394	152 (38.6%)	85 (21.6%)	55 (14.0%)	53 (13.5%)	29 (7.4%)	55 (14.0%)	12 (3.0%)	24 (6.1%)	8 (2.0%)	7 (1.8%)
Area	South China	1,159	410 (35.4%)	141 (12.2%)	126 (10.9%)	65 (5.6%)	30 (2.6%)	45 (3.9%)	33 (2.8%)	16 (1.4%)	6 (0.5%)	7 (0.6%)
	North China	1,333	353 (26.5%)	228 (17.1%)	183 (13.7%)	78 (5.9%)	109 (8.2%)	77 (5.8%)	42 (3.2%)	46 (3.5%)	51 (3.8%)	10 (0.8%)
Age	<6 months	685	270 (39.4%)	101 (14.7%)	77 (11.2%)	68 (9.9%)	60 (8.8%)	48 (7.0%)	40 (5.8%)	22 (3.2%)	16 (2.3%)	4 (0.6%)
	6 ~ 12 months	129	39 (30.2%)	12 (9.3%)	6 (4.7%)	5 (3.9%)	5 (3.9%)	4 (3.1%)	2 (1.6%)	3 (2.3%)	4 (3.1%)	0 (0.0%)
	>12 months	267	44 (16.5%)	19 (7.1%)	4 (1.5%)	4 (1.5%)	9 (3.4%)	15 (5.6%)	3 (1.1%)	5 (1.9%)	1 (0.4%)	0 (0.0%)
Vaccine	No	129	85 (65.9%)	10 (7.8%)	12 (9.3%)	6 (4.7%)	/	/	/	7 (5.4%)	2 (1.6%)	/
	Yes	417	84 (20.1%)	18 (4.3%)	9 (2.2%)	10 (2.4%)	/	/	/	20 (4.8%)	3 (0.7%)	/

/: not applicable, as analysis was limited to viruses included in common vaccines (CDV, CPV-2, CAV, CPIV, CCoV).

**Table 3:** Chi-square analysis of factors associated with prevalence of canine viral infections.

Factors	Heterogeneity	CPV-2	CCoV	CDV	CPIV	CHV-1	CIV	CRCoV	CAV-2	CAV-1	CRV
Season	*χ* ^2^	73.340	59.933	23.265	54.136	26.198	108.541	17.020	28.326	5.000	8.967
	*p*-value	<0.001	<0.001	<0.001	<0.001	<0.001	<0.001	0.001	<0.001	0.172	0.030
Area	*χ* ^2^	23.084	11.987	4.659	0.068	36.765	4.775	0.196	10.954	30.359	0.196
	*p*-value	<0.001	0.001	0.031	0.795	<0.001	0.029	0.658	0.001	<0.001	0.658
Age	*χ* ^2^	46.350	11.585	26.926	22.966	10.773	3.055	13.215	1.386	4.981	2.321
	*p*-value	<0.001	0.003	<0.001	<0.001	0.005	0.217	0.001	0.500	0.086	0.313
Vaccine	*χ* ^2^	30.359	2.390	13.597	1.758	/	/	/	0.083	0.750	/
	*p*-value	<0.001	0.122	<0.001	0.185	/	/	/	0.773	0.387	/

/: not applicable, as analysis was limited to viruses included in common vaccines (CDV, CPV-2, CAV, CPIV, CCoV).

### 3.3 Correlation between geographical location and ten viral infections

In the 1,159 dogs from South China, the prevalence of CPV-2, CCoV, CDV, CPIV, CHV-1, CIV, CRCoV, CAV-2, CAV-1, and CRV was 35.4, 12.2, 10.9, 5.6, 2.6, 3.9, 2.8, 1.4, 0.5, and 0.6%, respectively. In contrast, in North China, the prevalence was 26.5, 17.1, 13.7, 5.9, 8.2, 5.8, 3.2, 3.5, 3.8, and 0.8%, respectively (Table 2). The positive rates of CCoV, CDV, CHV-1, CIV, CAV-2, and CAV-1 in dogs from North China were significantly higher than those from South China. Conversely, the CPV-2 positive rate was significantly higher in dogs from South China than those from North China. No significant differences were found in the positive rates of CPIV, CRCoV, and CRV between South and North China (Table 3).

### 3.4 Correlations between the age of dogs and ten viral infections

To assess the rate of pathogen detection in relation to age, the dog population was divided into three categories: <6 months, 6–12 months, and >1 year old. There are detailed age records for 1,081 dogs, of which 685 under 6 months old, 129 between 6 and 12 months old, and 267 over 12 months old (Table 2). Young dogs (aged <6 months) were more commonly infected with CPV-2, CCoV, CDV, CPIV, CHV-1, and CRCoV compared to the other age groups. No significant association was found between age and the detection of CIV, CAV-2, CAV-1, or CRV (Table 3). The positive detection of CRV was observed exclusively in dogs aged <6 months.

### 3.5 Correlation between dog vaccination and ten viral infections

In China, commercially available vaccines for dogs typically include antigens for CDV, CPV-2, CAV, CPIV, and CCoV. Therefore, our analysis of the correlation between pathogen prevalence and immunization status was limited to these five viruses. The immunization status of 546 dogs was documented in detail, with 417 dogs having been vaccinated and 129 that never received vaccination. The positive rates of CPV-2, CCoV, CDV, CPIV, CAV-2, and CAV-1 in unvaccinated dogs were 65.9, 7.8, 9.3, 4.7, 5.4, and 1.6%, respectively. Meanwhile, the rates in vaccinated dogs were 20.1, 4.3, 2.2, 2.4, 4.8, and 0.7% (Table 2). The positive rates for CPV-2 and CDV in unvaccinated dogs were significantly higher than those in vaccinated dogs. There was no significant difference in the positive rate of CCoV, CPIV, CAV-2, and CAV-1 between vaccinated dogs and unvaccinated dogs (Table 3).

### 3.6 Antibody detection results for CDV, CPV-2, CAV-1, CAV-2, and CPIV in canine serum

504 serum samples of dogs were screened for antibodies against CPV-2, CDV, CAV-1, CAV-2, and CPIV. Antibodies against CPV-2 were detected by HI assay. CDV, CAV-1, CAV-2 and CPIV antibodies were detected by virus neutralization assay. The protective titers for CPV-2, CDV, CAV-1, CAV-2 and CPIV were 1:80, 1:32, 1:16, 1:16, and 1:16, respectively (24, 25). Of the 504 serum samples, 398 serum samples of dogs were vaccinated with modified-live CDV- CPV-2- CAV-2-CPIVvaccine, 15 serum samples of dogs have not been vaccinated, and the vaccination status of 91 serum samples were unknown. As shown in Table 4, the protective rate of CPV-2, CDV, CAV-1, CAV-2 and CPIV were 76.9% (306/398), 72.1% (287/398), 84.4% (336/398), 85.7% (341/398) and 49.0% (195/398) for the dogs had been vaccinated. Seroprevalence in unvaccinated dogs were 93.3% (14/15), 86.7% (13/15), 33.3% (5/15), 33.3% (5/15) and 53.3% (8/15) for CPV-2, CDV, CAV-1, CAV-2 and CPIV, respectively, (Table 4).

**Table 4:** The seroprotective rate of CPV-2, CDV, CAV-1, CAV-2 and CPIV in vaccinated and unvaccinated dogs.

Group	No.	No. of protective dogs (seroprotective rate %)				
		CPV-2	CDV	CAV-1	CAV-2	CPIV
Vaccinated	398	306 (76.9%)	287 (72.1%)	336 (84.4%)	341 (85.7%)	195 (49.0%)
Unvaccinated	15	14 (93.3%)	13 (86.7%)	5 (33.3%)	5 (33.3%)	8 (53.3%)

### 3.7 Phylogenetic analysis of CPV-2 and CPIV

**Figure 5:**
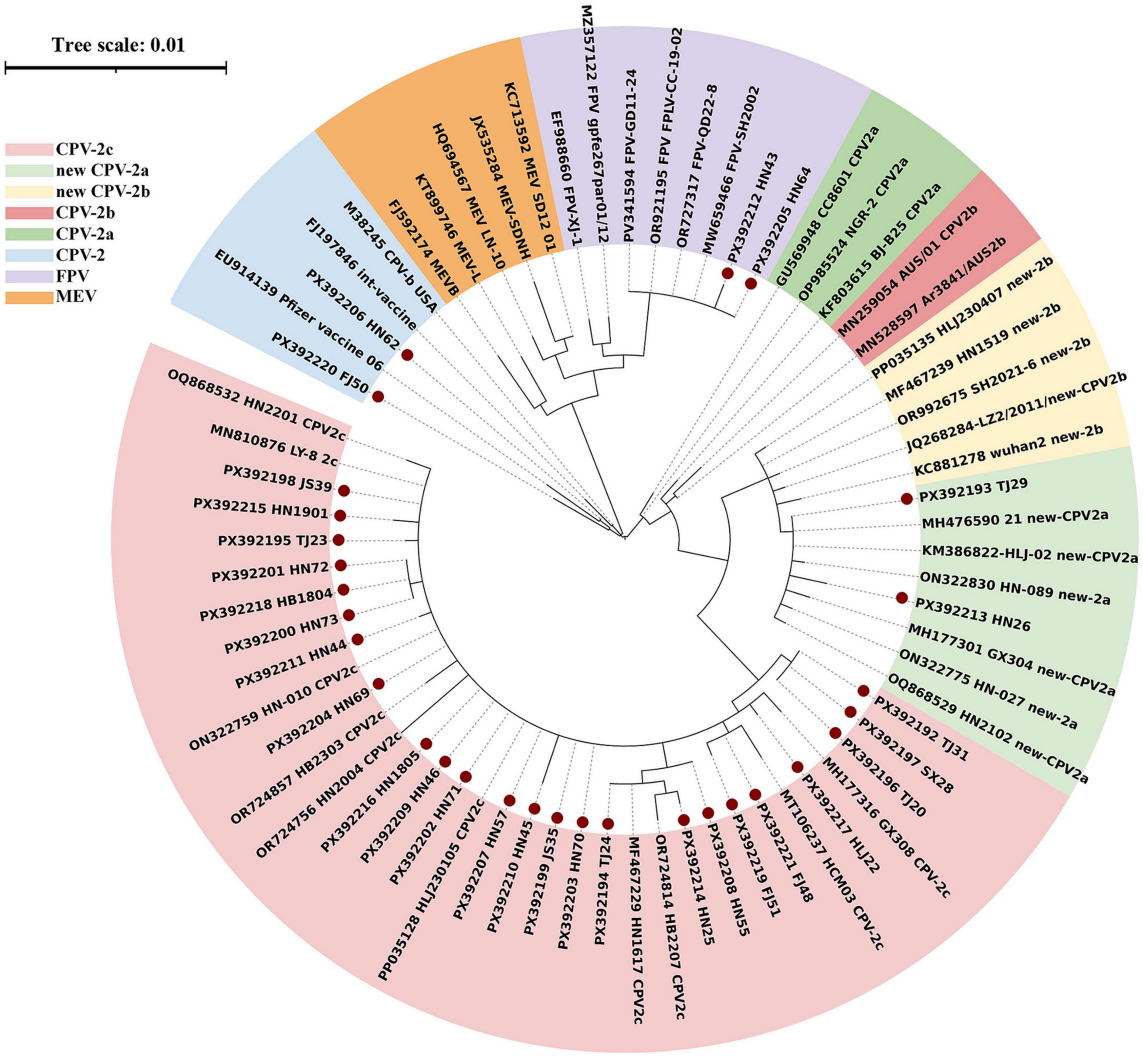
Phylogenetic tree of CPV-2 based on VP2 gene. Red circles indicate CPVs in this study.

To identify the genotype of CPV-2, the VP2 gene was sequenced in samples from 30 dogs. As shown in Figure 5, 24 CPVs were typed as CPV-2c genotype, 2 as new CPV-2a genotype, 2 as feline panleukopenia virus (FPV) and 2 as CPV-2 genotype (vaccine strain). CPV-2c was the most common genotype in the tested samples, accounting for 80.0% of 30 samples. New CPV-2a strains remained detectable at a frequency of 6.7%, whereas CPV-2b and the new CPV-2b variant were absent. Two samples identified as CPV-2 genotype also tested positive for CCoV, suggesting viral shedding after vaccination. Notably, two strains were identified as FPV, characterized by the signature residues 80Lys, 93Lys, 103Val, 323Asp, 564Asn, and 568Ala (25). All VP2 gene sequences have been deposited in GenBank under accession numbers PX392192 to PX392221.

**Figure 6:**
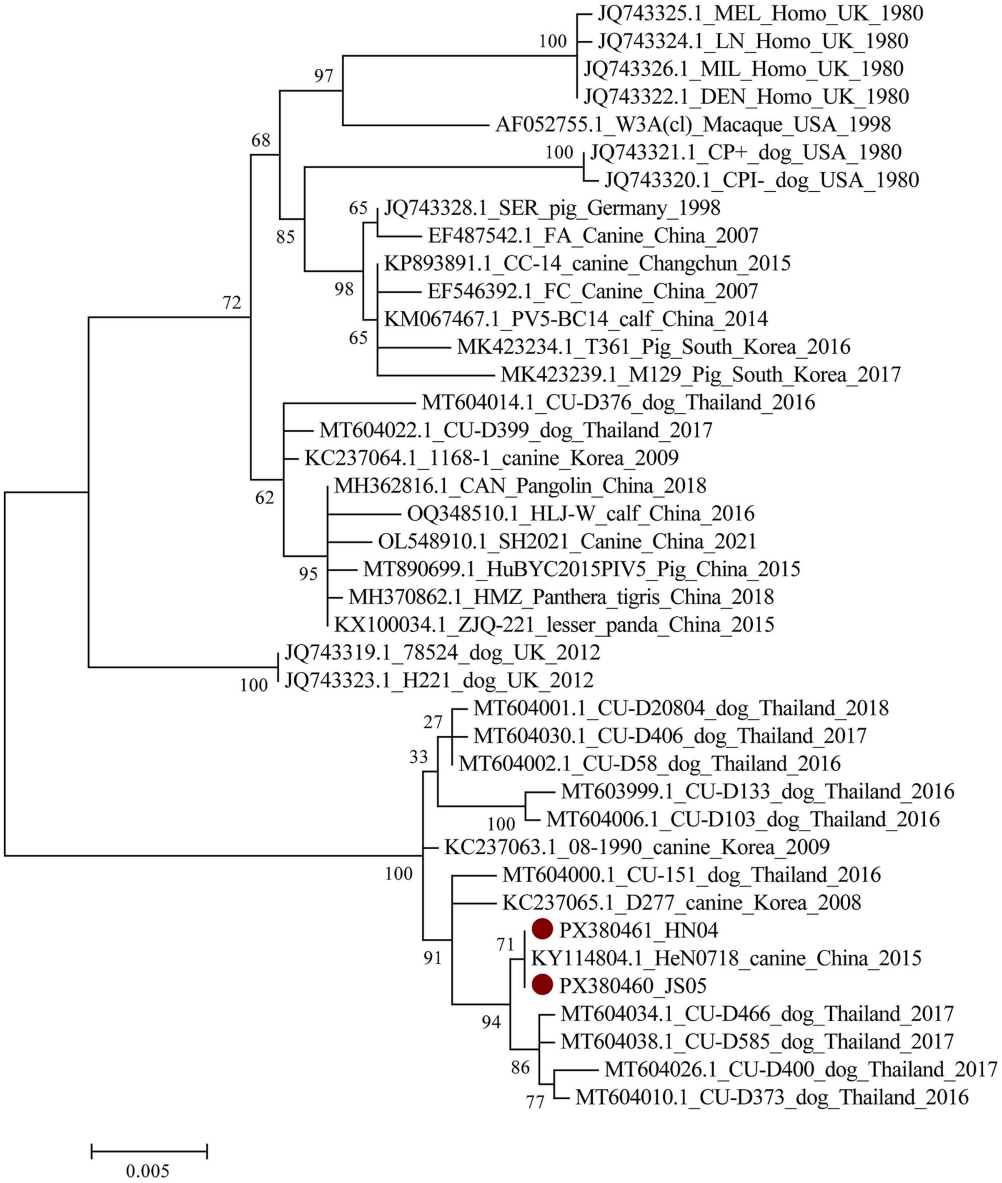
Phylogenetic analysis of CPIVs based on F gene sequences. Red circles indicate CPIVs in this study.

The F genes of currently circulating CPIV strains in China were sequenced and analyzed. The results showed that two CPIV strains were phylogenetically distant from strains circulating in the United States and the United Kingdom, yet clustered within the same clade as CPIV strains from Thailand, South Korea, and China (Figure 6). Unique amino acids of CPIVs in the distinct clade were observed at V104A, R237E, V290A, R299Q, I428V for F protein. The results revealed that these amino acids may be specific sites of canine-derived PIV. The F gene sequences have been deposited in GenBank under accession numbers PX380460 and PX380461.

## 4 Discussion

To understand viral infections in dogs in China, the present study determined the prevalence of ten pathogens, including CPV-2, CCoV, CDV, CPIV, CHV-1, CIV, CRCoV, CAV-2, CAV-1, and CRV. Recent epidemiological studies have provided data on infection rates and epidemic characteristics of various viruses in the region. However, a larger sample size is needed to minimize sampling error and increase the reliability of these findings. Thus, 2,492 dog samples from 22 provinces were investigated between 2018 and 2024. Of these, 49.6% (1,236/2,492) percentage of dogs were positive for one or more of the ten pathogens, and 57.8% (714/1,236) of dogs were mixed infections. Three or more simultaneous infections occur at a rate of 34.1%. The high prevalence of co-infection may increase disease severity and highlights the complexity of the canine viral infection in China (26, 27). CPV-2 and CCoV are common, globally distributed infections in dogs and pose significant threats to wildlife (2, 28), and they are both significant enteric pathogens primarily causing diarrhea (29, 30). Analysis of 2,492 samples revealed a high prevalence of CPV-2 (30.6%) and CCoV (14.8%), consistent with previously reported ranges in China of 5.9 to 74.1% for CPV-2 (29, 31–33), and 8.43 to 33% for CCoV (34–36). Canine infectious respiratory disease complex is associated with several viruses, including CDV, CPIV, CHV-1, CIV, CRCoV, and CAV-2. Among these, the prevalence of CDV in this study (12.4%) was the highest, aligning with rates previously reported in other Chinese cities that ranged from 10.0 to 24.9% (37–39). While the prevalence of CPIV and CRCoV in our study was lower than previously reported in China (34, 40, 41). This lower prevalence may reflect regional or temporal variations. Notably, CHV-1 and CRV prevalence in China have not been previously reported, our findings contribute to establishing a more comprehensive epidemiological dataset. Differences in prevalence are likely influenced by sample background and geographical location. Therefore, including samples from multiple regions with varied backgrounds is essential to improve the accuracy of future studies.

The survey of canine samples revealed correlations between prevalence and factors such as season, age, and geographical location. A distinct seasonal pattern was observed. Gastrointestinal pathogens such as CPV peaked in spring and winter. Whereas respiratory pathogens like CPIV, CIV, and CAV-2 were more prevalent in winter. This trend is likely attributable to cold exposure, which has been shown to impair the innate immune defenses of the upper respiratory tract mucosa, thereby reducing barriers to microbial invasion and facilitating pathogen entry (42). Geographically, CPV-2 showed a higher prevalence in southern China, likely due to the warm and humid climate that facilitates viral persistence in the environment. In comparison, higher prevalence rates of CCoV, CDV, CHV-1, CIV, CAV-2, and CAV-1, were observed in northern China. The cold and dry conditions in northern China may enhance the environmental stability and transmission of these viruses. These findings suggest that climatic conditions play a critical role in shaping the epidemiological patterns of canine viral infections in China. Moreover, rising temperatures, increased evaporation, and altered precipitation patterns associated with global warming (43) may influence the environmental persistence and seasonal transmission of several canine viruses in China. Long-term surveillance is therefore crucial to monitor these potential shifts.

The age distribution of canine viral infections in China shows distinct patterns, with variations observed across different pathogens. The highest prevalence rates were found in dogs younger than 6 months for CPV-2, CCoV, CDV, CPIV, CHV-1, and CRCoV. This trend likely reflects the immature immune systems of puppies and the decline of maternal antibodies, which may not provide adequate protection against these pathogens (44). Understanding these age-related differences in viral prevalence is essential for developing targeted vaccination and prevention strategies, with particular emphasis on high-risk age groups for specific pathogens.

The relationship between immunization status and the prevalence of major canine viruses in China is noteworthy. Vaccination significantly reduced the prevalence of CPV-2 and CDV. However, CCoV, CPIV, CAV-2, and CAV-1 showed no significant differences in prevalence between vaccinated and unvaccinated dogs. For CPIV, the seroprotection rate in vaccinated dogs was 49.0%, demonstrating suboptimal immunogenicity of the CPIV vaccine component. Sequencing of the CPIV F gene from two dogs revealed that the strains clustered more closely with isolates from Thailand and South Korea, rather than with strains from the United States and the United Kingdom, which form the basis of the vaccines currently used in China. Distinct amino acid substitutions were identified, suggesting the emergence of a potential Asian lineage.

For CPV, CPV-2c genotype was the most common genotype in this study, which aligns with the increasing prevalence of CPV-2c genotype in China in recent years (from 22.0% in 2015–2016 to 89.83% in 2020–2023) (23, 45). The vaccine strains of CPV-2 used in China are CPV-2 genotype, which may account for the high prevalence of CPV-2 in China. Notably, FPV was also detected in two dogs—one with diarrhea and another with diarrhea and vomiting, the latter also positive for CCoV, which supporting previous reports of FPV in Dogs from Egypt (46), Pakistan (47), Vietnam (48), Thailand (49) and China (50). These molecular differences may contribute to the reduced protective efficacy observed in vaccinated dogs and underscore the urgent need to update CPIV and CPV vaccine strains in China.

Several limitations should be acknowledged. First, the assessment of vaccine efficacy was limited to five viruses (CDV, CPV-2, CAV, CPIV, and CCoV) as these represent the only antigens included in standard canine vaccines in China. Consequently, associations between vaccination status and infections with other pathogens—such as CHV-1, CIV, CRCoV, and CRV—were not evaluated. Second, vaccination records were unavailable for 1,946 of 2,492 dogs (78.1%), resulting in an unbalanced comparison (Table 2) may overestimate vaccine effectiveness if animals lacking records were more likely to be unvaccinated. Third, missing age data for 54% of the cohort limited the ability to detect age-specific differences, particularly for viruses with low prevalence, such as CRV.

## 5 Conclusion

This study offers comprehensive data on the prevalence and distribution of ten canine viral pathogens in China from 2018 to 2024. The high prevalence of CPV-2, CCoV, and CDV, along with significant seasonal and geographical variations, underscores the complexity of canine viral infections. The differences in prevalence between vaccinated and unvaccinated dogs, genetic mutations in prevalent strains and the low seroprotective rate in vaccinated dogs highlight the importance of vaccination, while also pointing to potential limitations in the effectiveness of current vaccines. Collectively, our findings emphasize the need to improve immunization strategies and develop new vaccines to address the evolving epidemiological patterns of canine viral infections in China.

## Data Availability

The datasets presented in this study can be found in online repositories. The names of the repository/repositories and accession number(s) can be found in the article/supplementary material.

## Footnotes

1
http://itol.embl.de/
